# Nutritional Knowledge, Attitude, and Practices among HIV-positive Individuals in India

**DOI:** 10.3329/jhpn.v31i2.16383

**Published:** 2013-06

**Authors:** Deepika Anand, Seema Puri

**Affiliations:** Department of Food and Nutrition, Institute of Home Economics, University of Delhi, India

**Keywords:** HIV/AIDS, Knowledge, attitude and practices, Nutrition, India

## Abstract

This descriptive study investigated the nutrition-related knowledge, attitude, and practices (KAP) among people living with HIV/AIDS (PLHIV) in India. Data relating to nutritional KAP and sociodemographic profile were gathered from a sample of 400 PLHIV from New Delhi, India, using preset multiple-choice questionnaire. The knowledge on HIV/AIDS was low; nutritional knowledge was moderate as 80% of respondents could answer 4 out of 7 questions correctly. The attitude toward disease and food was positive but the application of nutritional knowledge was lacking as indicated by the moderate practice score of 8.1±2.3 out of a total score of 15. There were no significant differences in scores between genders. The PLHIV had knowledge about importance of nutrition during infection, had positive attitude toward the disease and the importance of nutrition during the course of the disease but translation of this knowledge into practice was low. Thus, there is a need for continuous interventions primarily aiming at behaviour change to convert knowledge into healthy dietary practices.

## INTRODUCTION

India is home to 1.2 billion people and is the second-most populous country in the world. Human Immunodeficiency virus (HIV) in India is mainly spread through heterosexual intercourse. The presence of ulcerative sexually transmitted diseases (STDs), irregular use of condoms, frequency of sexual contact, and age at sexual initiation are some of the factors affecting it. According to recent estimates of the National AIDS Control Organization (NACO), India had 2.2 million HIV-positive persons in 2008, with an estimated HIV prevalence of 0.29% among adults (1).

Importance of nutrition is well-established in HIV infection (2-4). Poor nutritional status is a strong predictor of mortality. Even after controlling for CD4+ cell counts, a weight loss of >66% of ideal body-weight was linked to the timing of death in AIDS patients (5-7). Although there have been a number of studies assessing the knowledge, attitude, and practices relating to antiretroviral therapy (ART), not many studies have assessed these keeping nutrition as their main focus (8-10). This study was, therefore, undertaken to assess the nutrition-related knowledge, attitude, and practices of PLHIV.

## MATERIALS AND METHODS

Data for this cross-sectional descriptive study were collected from a total of 400 PLHIV (245 male, 144 female, and 11 transgender) registered at the ART centre of Guru Teg Bahadur Hospital, Shahadra, Delhi, India. The permission to carry out the study was obtained for a period of 12 weeks. Taking this stipulated timeframe, data were collected from these 400 PLHIV. The inclusion criteria for the study were to enrol PLHIV who attended the ART centre over a period of three months, were more than 21 years of age, had the record of CD4 estimations within the last 30 days from the date of data collection, and agreed to answer the questions relating to nutrition. Infants, children, adolescents, and pregnant/lactating mothers, those not registered at the centre, and those who refused to participate in the study were excluded.

Ethical approval was obtained from the Institutional Ethics Committee of the Institute of Home Economics, University of Delhi, India. Details of the study procedures were given on the volunteer's information sheet (in English and Hindi language). The features pertaining to benefits, confidentiality, and voluntary participation were explained, and written informed consent was obtained from all the subjects.

Sociodemographic information was gathered using a preset multiple-choice questionnaire. A close-ended, pretested questionnaire comprising three sections: knowledge (15 multiple-choice questions), attitude (15 statements), and practices (15 multiple-choice questions) was used in eliciting KAP information.

### Scoring

The scoring mechanism for each section was developed by the researchers. For knowledge, each correct answer was given a score of 1 while each wrong answer was given a score of 0. Each question in the knowledge section only had one correct answer and three wrong answers. This way, a respondent could score a maximum of 15 and a minimum of 0 in this section. For attitude, a score of 1 was given to ‘disagree’, a score of 2 was given to ‘don't know’, and a score of 3 was given to ‘agree’. There were eight negatively-framed statements for which reverse scoring was done which means a score of 1 was given to ‘agree’, 2 to ‘don't know’, and 3 to ‘disagree’. This way, a respondent could score a maximum of 45 and a minimum of 15 in the attitude section. For practices, each ‘yes’ response received a score of 1 while each ‘no’ received a score of 0. Therefore, a respondent could score a maximum of 15 and a minimum of 0 in the practice section. The final KAP score was calculated by summing up the scores of the knowledge, attitude and practices sections individually. The total KAP score ranged from 15 to 75. The higher the score, the better were the respondent's nutrition-related knowledge, attitude, and practice. The scores were also classified into poor, average, and good scores, ranges for which are shown in Table 1.

**Table 1.** Scoring and classification of KAP

	Knowledge	Attitude	Practices	KAP
Scoring	1 for correct answer 0 for incorrect answer	1=Disagree 2=Don't know 3=Agree	1=Yes 0=No	Knowledge score + Attitude score + Practices score
Range	0-15	15-45	0-15	15-75
Classification	Poor: 0-5 Average: 6-10 Good: 11-15	Poor: 15-25 Average: 26-35 Good: 36-45	Poor: 0-5 Average: 6-10 Good: 11-15	Poor: 15-35 Average: 36-55 Good: 56-75

All statistical analyses were carried out in STATA (version 9), which included frequency count and percentages. Descriptive statistics were used for giving a clear picture of sociodemographic variables.

## RESULTS

### Sociodemographic profile

The complete sociodemographic profile of the study sample is shown in Table 2. Majority of them (82.7%) were 21-40 years old. Around 31% had no education; 15% had primary-level education; 44% had secondary and 10% had college-level or higher education. Heterosexual contact, including unsafe sexual practices, was the major route for HIV transmission (76.5%) in the study sample. In terms of marital status, 15.5% of the participants were widowed, 13% were single, and around 1% were separated or divorced. The rest (70.75%) were married. The majority of the participants (84.76%) followed Hinduism as their religion, and 12.28% followed Islam. Majority of the subjects (63%) belonged to nuclear families. At the time of interview, 38% of the subjects were unemployed, 29% were in some kind of business, 20.5% had salaried jobs, 9% were involved in heavy work, like cultivation or construction work, and 3.5% reported to be students or domestic helpers. Around 50% of the subjects reported their annual family income to be less than Rs. 30,000. Taking into consideration the average size of the family as 4 in the present study, 50% of the subjects had per-capita monthly income of less than Rs. 625.

**Table 2.** Sociodemographic information on PLHIV

Characteristics	Male n (%)	Female n (%)	Transgender n (%)	Total n (%)
Age (years)				
21–<41	203 (82.9)	120 (83.3)	8 (72.7)	331 (82.7)
41–59	42 (17.1)	24 (16.7)	3 (27.3)	69 (17.3)
Risk factor for acquiring HIV				
Sexual route	187 (76.3)	114 (79.2)	5 (45.5)	306 (76.5)
Injecting drug	8 (3.3)	1 (0.7)	5 (45.5)	14 (3.5)
Blood transfusion	13 (5.3)	15 (10.4)	0	28 (7)
Mother-to-child transmission	2 (0.8)	0	0	2 (0.5)
Probable unsafe injection	14 (5.7)	1 (0.7)	1 (9.0)	16 (4)
Unknown	21 (8.6)	13 (9.0)	0	34 (8.5)
Present treatment status				
On ART	166 (67.8)	89 (61.8)	7 (63.6)	262 (65.5)
Not on ART	79 (32.2)	55 (38.2)	4 (36.4)	138 (34.5)
Education				
No education	52 (21.3)	62 (43.1)	9 (81.8)	123 (30.7)
Primary	42 (17.1)	18 (12.5)	1 (9.1)	61 (15.3)
Secondary	128 (52.2)	47 (32.6)	1 (9.1)	176 (44)
College and above	23 (9.4)	17 (11.8)	0	40 (10)
Marital status				
Single	39 (15.9)	5 (3.5)	8 (72.7)	52 (13)
Married	191 (78)	89 (61.8)	3 (27.3)	283 (70.7)
Divorced/Separated	3 (1.2)	0	0	3 (0.8)
Widowed	12 (4.9)	50 (34.7)	0	62 (15.5)
Religion				
Hindu	208 (85.2)	126 (87.5)	5 (45.4)	339 (84.8)
Muslim	30 (12.3)	13 (9.0)	6 (54.6)	49 (12.4)
Others	6 (2.5)	5 (3.5)	0	11 (2.8)

#### Knowledge

A composite score in the knowledge section was computed as the number of correct responses to the questions (range 0-15). The overall mean±SD was 8.3±2.2, which indicated that, on an average, the respondents knew the correct answer to 55% of the questions.

In the knowledge section (Table 3), first eight questions pertained specifically to HIV/AIDS, eliciting participants’ knowledge on modes of HIV transmission, risk, symptoms, and side-effects of ART. Out of eight questions, only three were answered correctly by more than 50% of the respondents, indicating limited knowledge on HIV/AIDS. Only around 40% could identify the correct modes of transmission of HIV, symptoms of HIV/AIDS, and side-effects of ARV medicines. Three-fourths (75%) of the respondents knew that ARV medicines are given to HIV-positive people, and around 65% could identify that low CD4 count confirms the HIV-positive status.

**Table 3.** Percentage of respondents who answered each of the knowledge-related questions correctly

Question	N (%)
Modes of transmission of HIV/AIDS	151 (37.8)
Who are at the greatest risk of acquiring HIV infection?	205 (51.3)
Common symptoms of HIV infection	155 (38.8)
HIV status is confirmed by which biological condition?	261 (65.3)
What component of body is adversely affected by HIV infection?	124 (31)
Which of the following daily activities lead to the spread of HIV infection?	185 (46.3)
ARV medicines are given to whom?	293 (73.3)
Common side-effects of ARV medicines	157 (39.3)
HIV infection increases the body's requirement for which nutrients?	101 (25.3)
PLHIV should follow which healthy practices in their daily routine?	325 (81.3)
PLHIV should not follow which of the following dietary practices?	295 (73.8)
Which of the following have deleterious effect on health?	359 (89.8)
Which of the following are excellent sources of protein?	94 (23.5)
Which of the following is the safest way of drinking water?	269 (67.3)
What food-items should not be consumed, along with ARV medicines?	360 (90)

The overall nutrition-related knowledge was good among the respondents as approximately 80% of the respondents could answer more than 50% of the nutrition-related questions correctly. The specific nutrient-related information was lacking as only around 24% of the respondents could not identify the correct nutrients required during the infection.

Overall, knowledge score (mean±SD) of the subjects was 8.3±2.2, which lied in the moderate category. Classification of subjects on the basis of poor (0-5), average (6-10), and good (11-15) knowledge scores indicated 12%, 71.2%, and 16.8% of subjects in the respective categories.

#### Attitude

The responses to the statements framed to know the attitude of PLHIV were marked on three pointer scale (Agree, Don't know, and Disagree) as shown in Table 4.

**Table 4.** Attitude of the respondents

Statement	Agree n (%)	Don't know n (%)	Disagree n (%)
Males and females have the same probability of acquiring HIV infection	137 (34.3)	52 (13)	211 (52.7)
HIV/AIDS affects both rich and poor	324 (81)	27 (6.8)	49 (12.2)
Children born to HIV-infected mothers should not receive immunization	138 (34.5)	130 (32.5)	132 (33)
TB always accompanies HIV/AIDS	187 (46.8)	114 (28.5)	99 (24.7)
Opportunistic infections that accompany HIV/AIDS occur due to weakened immunity	211 (52.8)	52 (13)	137 (34.2)
Non-vegetarian food is more nutritious than vegetarian food	132 (33)	103 (25.8)	165 (41.2)
Light daily exercise does not play an important role in the management of HIV/AIDS	53 (13.3)	31 (7.7)	316 (79)
Smoking does not cause any additional harm to the body	67 (16.8)	50 (12.5)	283 (70.7)
A nutritious diet includes expensive food	134 (33.5)	84 (21)	182 (45.5)
Maintaining self-hygiene is as important as eating nutritious food	290 (72.5)	49 (12.3)	61 (15.2)
Diet and nutrition play a very important role in maintaining good health in PLHIV	291 (72.8)	45 (11.2)	64 (16)
Cooked vegetables are more microbiologically safe but less nutritious than raw vegetables	209 (52.3)	59 (14.7)	132 (33)
Drugs, like ARVs, are associated with side-effects that may reduce food intake	191 (47.8)	83 (20.7)	126 (31.5)
One should not eat during the phase of diarrhoea	108 (27)	105 (26.3)	187 (46.7)
Food cannot be a source of infection ever	161 (40.3)	70 (17.5)	169 (42.2)

The overall attitude score for the sample was 34.4±3.7. A large percentage of respondents believed that HIV/AIDS affects both rich and poor (81%), opportunistic infections essentially occur due to weakened immunity (53%), and TB was believed to be always associated with HIV/AIDS (47%). With respect to gender, a negative attitude prevailed as only around 35% believed that HIV infection is common to both males and females. There was not much clarity about the issue of child immunization in the present sample.

Ten statements were framed to see the perception of PLHIV toward nutrition and HIV/AIDS. The statements covered the topics, like healthy food choices, healthy dietary practices, and food interaction, during the disease. There were four negatively-framed statements, which were reversed for the purpose of scoring.

Majority of the respondents (approximately 75%) believed that exercise helps in the management of the disease, smoking is harmful for health, self-hygiene is very important, and good nutrition is important for PLHIV. However, the attitude of respondents toward issues, like making healthy food choices, cooking of vegetables, managing ART side-effects, and food safety, was weak as the strength in their response was neither positive nor negative.

Classification of subjects on the basis of scores obtained on attitude section indicated 38% in good (36-45), 60.8% in average (26-35), and only 1.2% in poor (15-25) scoring on this section.

#### Practices

The information on the practices was elicited by the responses with ‘yes’ or ‘no’. The percentages of the respondents who indulged in healthy dietary practices are shown in Table 5.

**Table 5.** Percentage of respondents indulged in healthy dietary practices

Question	N (%)
Do you indulge in any type of exercise or light physical activity daily?	65 (16.3)
Do you drink filtered water or boiled water before drinking?	118 (29.5)
Do you include snacks in between your main meals?	227 (56.8)
Do you consume seasonal fruits or vegetables daily in your diet?	323 (80.8)
Do you consume milk or milk products, like paneer, curd, etc. daily?	199 (49.8)
Do you drink at least 8-10 glasses of water every day?	212 (53)
Do you continue to eat food during the period of illnesses?	243 (60.8)
Is there any reduction in the alcohol consumption or in the number of cigarettes smoked in the past six months?	322 (80.5)
Do you take medicines after meals?	381 (95.3)
Do you indulge in stress-relieving practices, like yoga, prayers, etc.?	82 (20.5)
Do you keep a track of your weight?	251 (62.8)
Do you consume fried foods or add oil on dishes before consuming?	233 (58.3)
Do you make your meals nutritious by adding nuts, jaggery, etc.?	153 (38.3)
Are you taking any kind of vitamin or mineral supplements?	56 (14)
Do you wash fruits and vegetables before consumption?	358 (89.5)

The overall score in the practices session ranged from 0 to 15, and the mean±SD score of the sample was 8.1±2.3, which again lied in the moderate category (6-10).

Questions relating to practices elicited very interesting information. Participants indulged in a number of good practices, like taking medicines after meals (95.3%), including fruits and vegetables in their daily diet (80%), washing fruits and vegetables before consuming (89.5%), and also around 80% reported to have reduced the intake of alcohol and cigarettes (80.5%) in the past six months. The practices, like daily consumption of milk or milk products, needed attention as only 50% of respondents reported daily consumption of these products. Consumption of snacks not essentially healthy and fried food was high as reported by approximately 60% of respondents. Indulgence in any kind of exercise or stress-relieving practices was very low among the sample. Also, consumption of filtered or boiled water or vitamin/mineral supplements was reported by a very small proportion of the sample (30% and 14% respectively). Thus, there were areas identified (healthy and safe food choices and practices, healthy snacking, regular health monitoring, and regular exercise), which needed counselling to bring about behaviour change and to inculcate good dietary habits among PLHIV.

#### Total score in KAP

The mean scores of the sample on knowledge, attitude, practices, and total KAP are shown in Table 6. The overall scores in knowledge, attitude, practices, and total KAP lie in the medium category (6-10 for knowledge and practices, 26-35 for attitude, and 36-55 for total KAP) for the entire sample.

**Table 6.** Mean±SD KAP scores of the sample

Section	Score range	Male (n=245)	Female (n=144)	Transgender (n=11)	Total (n=400)
Knowledge	0-15	8.5±2.2	8.1±2.3	9.4±2.1	8.3±2.2
Attitude	15-45	34.6±3.6	34.3±3.7	32.5±4.5	34.4±3.7
Practices	0-15	8.1±2.5	8.0±1.9	9±2.9	8.1±2.3
Total KAP	15-75	51.1±5.4	50.4±5.7	50.8±4.7	50.8±5.5

## DISCUSSION

Importance of nutrition in the care and treatment for PLHIV is well-established. The role of malnutrition in adversely affecting the treatment course is also well-documented (11). In India, little is known about the level of nutrition-related knowledge, attitude, and what the common nutritional practices are prevalent among PLHIV. This study was an attempt to elicit information on all these issues.

The results from the study indicated that the basic HIV-related knowledge was lacking among the subjects. However, their knowledge on the nutritional aspects was moderate. Repeated counselling sessions at the ART centre could be the reason for better knowledge scores among these subjects. The PLHIV in the present sample had positive attitude toward the disease and recognized the importance of nutrition in HIV. The study also identified a few areas where PLHIV did not have positive attitude wherein effective messages may be crafted and delivered to remove the negativity and to strengthen the belief that good and nutritious food is important for healthy living. Food safety and healthy food choices are the identified areas.

The PLHIV in the present study possessed knowledge about nutrition but did not score very high in the practices section. This indicates that the nutritional knowledge possessed by them was not applied in their day-to-day practices. The present study did not enquire about the reasons for not exercising, not boiling water before consumption, not making any efforts to improve the diet quality. However, there are previous studies which identified that failure of households to ensure the availability of food and access, low socioeconomic status, level of education, personal beliefs, availability of food, and low nutritional knowledge were the reasons for poor dietary practices (12-17). The need is to undertake interventions primarily aiming at bringing about behaviour change in the PLHIV for developing good and healthy nutritional practices.

## ACKNOWLEDGEMENTS

Authors would like to thank all the participants and the staff of Guru Teg Bahadur Hospital, New Delhi, for their cooperation.
